# Supplementary material for: Initiation of domiciliary care and nursing home admission following first hospitalization for heart failure, stroke, chronic obstructive pulmonary disease or cancer
Source: PLoS One. 2021 Aug 4;16(8):e0255364. doi: 10.1371/journal.pone.0255364 (PMC8336831; doi:10.1371/journal.pone.0255364)
Supplement: S1 Appendix — (DOCX) [file pone.0255364.s001.docx]

**Appendix 1 ICD-10 codes**

| **Comorbidity** | **ICD-10 codes** |
| --- | --- |
| Heart failure | I420, I426, I427, I428, I429, I50, I110, I130, I132 |
| Stroke | I60-64 |
| Chronic obstructive pulmonary disease | J42-J44 |
| Cancer | C00-C97 (if not C44) |
| Ischemic heart disease | I20-I25 |
| Atrial fibrillation | I48 |
| Diabetes | E10-14 |
| Chronic kidney disease | E102, E112, E132, E142, I120, N02-N08, N11, N12, N14, N18, N19, N26, N158-N160, N162-N164, N168, M300, M313, M319, M321B, Q612, Q613, Q615, Q619, T858, T859, Z992 |
| Dementia | F00-F03 |
| Depression | F31-34 |

ICD, International Classification of Diseases
