# Supplementary material for: Initiation of domiciliary care and nursing home admission following first hospitalization for heart failure, stroke, chronic obstructive pulmonary disease or cancer
Source: PLoS One. 2021 Aug 4;16(8):e0255364. doi: 10.1371/journal.pone.0255364 (PMC8336831; doi:10.1371/journal.pone.0255364)
Supplement: S2 Appendix — (DOCX) [file pone.0255364.s002.docx]

**Appendix 2. ATC classification codes**

| Pharmacotherapy | ATC codes |
| --- | --- |
| Glucose lowering drugs | A10 |
| Dementia | N06D |
| Depression | N06A |
| Antiplatelets | B01AC06, N02BA01, B01AC04, B01AC22, B01AC24, B01AC07 |
| Lipid-lowering drugs | C10 |
| Thiazides | C03A, C07B, C07D, C09XA52, C03EA01 |
| Loop diuretics | C03C, C03EB01, C03EB02 |
| Beta-blockers | C07, C09BX |
| Renin-angiotensin-system inhibitors | C09 |

ATC, Anatomical Therapeutic Chemical
